# Supplementary material for: Rapid turnover of life-cycle-related genes in the brown algae
Source: Genome Biol. 2019 Feb 14;20:35. doi: 10.1186/s13059-019-1630-6 (PMC6374913; doi:10.1186/s13059-019-1630-6)
Supplement: Supplementary file 3 — Protein alignments, corresponding Gblocks html file and CDS sequences for the lineage conserved gametophyte-biased genes. (ZIP 2109 kb) [file 13059_2019_1630_MOESM3_ESM.zip › Con_GA_alignments/Ec-27_006610.aa_ali.fasta-gb.htm]

Ec-27\_006610.aa.fasta.trans.aa\_ali.fasta


## Gblocks 0.91b Results

Processed file: **Ec-27\_006610.aa.fasta.trans.aa\_ali.fasta**  
Number of sequences: **2**  
Alignment assumed to be: **Protein**  
New number of positions: **215** (selected positions are underlined in blue)

```
                         10        20        30        40        50        60
                 =========+=========+=========+=========+=========+=========+
Ec-27_006610     --MATVSPRLHHDIERRLRGAYSRDLENRIHHNNRSSSVPAG--SFPNYHRNEAGRATTA
MSTRG.26984.1    XSMSMVSPRLHRDMNRRVRAAYSVDVD-----KHRCSSVPTASIRYQCSDQMDTADATRS
                   #######################                               ####


                         70        80        90       100       110       120
                 =========+=========+=========+=========+=========+=========+
Ec-27_006610     PSATGYGRSTARPRLMSSTKHIRVARSQSPSS------VPPPGSSFAGSDCCKPCYQRPV
MSTRG.26984.1    SGFRGYRASAIRSRLMSPTKHIRVARSEPPPSSAGRYLAEVEDRSHPRREASSRCAVGQA
                 ################################                      ######


                        130       140       150       160       170       180
                 =========+=========+=========+=========+=========+=========+
Ec-27_006610     GQDQQVDQAGRCEQREENLPPTTSRAVGASTTVPLSVNVETPLAGESSLSDADERLNVST
MSTRG.26984.1    KHDDPKGQRGDLIKQRALAPPTVSSTIGLSG-PPLEVNVKAPLSVEDGFLSAAE--NSTQ
                 ##########         ###########   #####################  ####


                        190       200       210       220       230       240
                 =========+=========+=========+=========+=========+=========+
Ec-27_006610     PSPATAAAEEEHALLSRERAGTPVS-EMGTPKKLLELIEDRDRALHLCLQYRCKLRNTEG
MSTRG.26984.1    SSPTTTAEGAGKVGLSRGQTEAPIILELQSPKKLMQVMEERDRAIHVCLQYRCKLKNTEG
                 #######################   ##################################


                        250       260       270       280       290       300
                 =========+=========+=========+=========+=========+=========+
Ec-27_006610     SLRAEERLASELKGQLREANAAGEATRKLFDQVMDKVRADEGRKIRQAASSREDDKSNAR
MSTRG.26984.1    SLRAEERLASDLKAQLREANAAGEATRKLFDQVMDKVRADEGRKLRQEVDN---------
                 ###############################################             


                        310       320       330       340       350       360
                 =========+=========+=========+=========+=========+=========+
Ec-27_006610     YAEDARKGRLQLTRARKDLRELWLERDELAKLAGQAIQGEQAGSKRIKKLELVVSSLREQ
MSTRG.26984.1    ------------------------------------------------------------
                                                                             


                        370       380       390       400       410       420
                 =========+=========+=========+=========+=========+=========+
Ec-27_006610     VAALALSEHRAAEEAARAEARAASAEAAEQDLRAAVEGIKTDFEALVHELRIREKQIRQM
MSTRG.26984.1    ------------------------------------------------------------
                                                                             


                        430       440       450       460       470       480
                 =========+=========+=========+=========+=========+=========+
Ec-27_006610     EAKNATLAVKSLPTSPTPRGHTRSRSPAETGNAEGDNQRVSPRTRRMTPESKGCVRVKEL
MSTRG.26984.1    ------------------------------------------------------------
                                                                             


                        490       500       510       520       530       540
                 =========+=========+=========+=========+=========+=========+
Ec-27_006610     RKSRNSRVDPSPARAKEATSLNTQALAHPQKRGKRGEEAGIPPGKNGRRSNAPPAAAVPS
MSTRG.26984.1    ------------------------------------------------------------
                                                                             


                        550       560       570       580       590       600
                 =========+=========+=========+=========+=========+=========+
Ec-27_006610     VVYPARKQLAPRKSEIPGRVMAMSYPRGVNRVTFMPSNTHVTTPPARVPASVPARETVRP
MSTRG.26984.1    ------------------------------------------------------------
                                                                             


                        610       620       630       640       650       660
                 =========+=========+=========+=========+=========+=========+
Ec-27_006610     VGPILEEGNCCMEEVEEEVEEEEDGGGEVCSAPTEVAGTVTDDGRRPEAQLDQSGEEKSG
MSTRG.26984.1    ------------------------------------------------------------
                                                                             


                        670       680       690       700       710       720
                 =========+=========+=========+=========+=========+=========+
Ec-27_006610     APETSPPSPGQDCAVPPQSPSPRHCRQPACTFSETAAPPASDPRPTVGMSNTTEVALADL
MSTRG.26984.1    ------------------------------------------------------------
                                                                             


                        730       740       750       760       770       780
                 =========+=========+=========+=========+=========+=========+
Ec-27_006610     GGAAPEDGVVGLGSAGGTERLTTDAETREGADNASAEGVPPPPLAQIASTGRFSGDDDEP
MSTRG.26984.1    ------------------------------------------------------------
                                                                             


                        790       800       810       820       830       840
                 =========+=========+=========+=========+=========+=========+
Ec-27_006610     GEEKSTPHGGKIEEAKSEAGHGIEATPEQPGLEEGRRDPDAAAFRPRLVEGVPVLKYGGR
MSTRG.26984.1    ------------------------------------------------------------
                                                                             


                        850       860       870       880       890       900
                 =========+=========+=========+=========+=========+=========+
Ec-27_006610     GKPKAKMLWVTPDLSEIFYTQAGRTKNSKKSSHMPLAGLCASKGRGDHPEPARSTTDDRC
MSTRG.26984.1    ------------------------------------------------------------
                                                                             


                        910       920       930       940       950       960
                 =========+=========+=========+=========+=========+=========+
Ec-27_006610     FALVQAADSLGHDNPHHKDKDGGGSESNGAGEDADEGGGGGGGSPAPGRRKWGIGGTLPA
MSTRG.26984.1    ------------------------------------------------------------
                                                                             


                        970       980       990      1000      1010      1020
                 =========+=========+=========+=========+=========+=========+
Ec-27_006610     NLVHGRRWRGGPGSAATTTGPTERLGASATSASGSAATAAAVGTGAGAAPQQHPAGATRE
MSTRG.26984.1    ------------------------------------------------------------
                                                                             


                       1030      1040      1050      1060      1070      1080
                 =========+=========+=========+=========+=========+=========+
Ec-27_006610     RMVLETTNAEECTALVDEINELVRRAGLQRGGGGSSARGRDLFDEWVRSAVGRDAQAVDP
MSTRG.26984.1    ------------------------------------------------------------
                                                                             


                       1090      1100
                 =========+=========+========
Ec-27_006610     GANGEAAEGAQEGRGSAGGDAPVALLAG
MSTRG.26984.1    ----------------------------
```

```
Parameters used
Minimum Number Of Sequences For A Conserved Position: 2
Minimum Number Of Sequences For A Flanking Position: 2
Maximum Number Of Contiguous Nonconserved Positions: 8
Minimum Length Of A Block: 10
Allowed Gap Positions: None
Use Similarity Matrices: Yes
```

```
Flank positions of the 7 selected block(s)
Flanks: [3  25]  [57  92]  [115  130]  [140  150]  [154  174]  [177  203]  [207  287]  

New number of positions in Ec-27_006610.aa.fasta.trans.aa_ali.fasta-gb:  215  (19% of the original 1108 positions)
```
